# Hippocampal subfield atrophy in relation to cerebrospinal fluid biomarkers and cognition in early Parkinson’s disease: a cross-sectional study

**DOI:** 10.1038/npjparkd.2015.30

**Published:** 2016-01-14

**Authors:** Ane Løvli Stav, Krisztina Kunszt Johansen, Eirik Auning, Lisa Flem Kalheim, Per Selnes, Atle Bjørnerud, Erik Hessen, Dag Aarsland, Tormod Fladby

**Affiliations:** 1 Department of Neurology, Akershus University Hospital, Lørenskog, Norway; 2 Faculty Division Akershus University Hospital, University of Oslo, Oslo, Norway; 3 Department of Geriatric Psychiatry, Akershus University Hospital, Lørenskog, Norway; 4 The Intervention Centre, Oslo University Hospital, Rikshospitalet, Oslo, Norway; 5 Department of Physics, University of Oslo, Oslo, Norway; 6 Department of Psychology, University of Oslo, Oslo, Norway; 7 Department of Neurobiology, Alzheimer’s Disease Research Centre, Care Sciences and Society, Karolinska Institutet, Stockholm, Sweden; 8 Centre for Age-Related Diseases, Stavanger University Hospital, Stavanger, Norway

## Abstract

Cognition is often affected early in Parkinson’s disease (PD). Lewy body and amyloid β (Aβ) pathology and cortical atrophy may be involved. The aim of this study was to examine whether medial temporal lobe structural changes may be linked to cerebrospinal fluid (CSF) biomarker levels and cognition in early PD. PD patients had smaller volumes of total hippocampus, presubiculum, subiculum, CA2–3, CA4-DG, and hippocampal tail compared with normal controls (NCs). In the PD group, lower CSF Aβ38 and 42 were significant predictors for thinner perirhinal cortex. Lower Aβ42 and smaller presubiculum and subiculum predicted poorer verbal learning and delayed verbal recall. Smaller total hippocampus, presubiculum and subiculum predicted poorer visuospatial copying. Lower Aβ38 and 40 and thinner perirhinal cortex predicted poorer delayed visual reproduction. In conclusion, smaller volumes of hippocampal subfields and subhippocampal cortex thickness linked to lower CSF Aβ levels may contribute to cognitive impairment in early PD. Thirty-three early PD patients (13 without, 5 with subjective, and 15 with mild cognitive impairment) and NC had 3 T magnetic resonance imaging (MRI) scans. The MRI scans were post processed for volumes of hippocampal subfields and entorhinal and perirhinal cortical thickness. Lumbar puncture for CSF biomarkers Aβ38, 40, 42, total tau, phosphorylated tau (Innogenetics), and total α-synuclein (Meso Scale Diagnostics) were performed. Multiple regression analyses were used for between-group comparisons of the MRI measurements in the NC and PD groups and for assessment of CSF biomarkers and neuropsychological tests in relation to morphometry in the PD group.

## Introduction

Cognitive impairment (CI) is a common non-motor feature in Parkinson’s disease (PD). Approximately, 25% of non-demented PD cases have mild CI (MCI), which may precede dementia.^1^ Research on the role of central nervous system structural affection and associated molecular mechanisms for cognitive decline in PD is needed for early diagnosis and intervention.

Imaging studies in PD have shown different patterns of magnetic resonance imaging (MRI) cortical gray matter atrophy. In PD without dementia, little or no regional thinning has been found in hippocampus, temporal, limbic, frontal, parietal, or occipital cortex, but there are more pronounced changes in PD-MCI and widespread atrophy is described in PD with dementia.^2–4^ Hippocampal atrophy has been associated with CI, especially poorer memory, in PD with and without dementia.^3^

Learning, memory, and visuospatial functions are related to hippocampus and subhippocampal regions (including entorhinal and perirhinal cortices, entorhinal cortex (ERC), and perirhinal cortex (PRC)) of the medial temporal lobe.^5,6^ The hippocampal subfields are in varying degree present within the head, body, and tail of the hippocampus.^7^ The hippocampus proper includes the dentate gyrus (DG) tightly connected to CA4, and the remaining subfields of cornu ammonis (CA3-1)^5^ filled by densely packed pyramidal cells^6^ similarly layered as the neocortex. As described in primate models, associative cortical input is transferred mainly through PRC and ERC, and processed through the hippocampal subfields by way of the highly plastic connections in the polysynaptic loop to CA1, subiculum, and presubiculum.^6^ These output structures project back to ERC, PRC and prefrontal, thalamic, parahippocampal, and retrosplenial regions.^5^

MCI and Alzheimer’s disease (AD) have been linked to medial temporal lobe affection including CA1-4, DG, subiculum, presubiculum, ERC, and PRC^8–12^ that are related to poorer memory functions.^9–11^ Selective vulnerability of different hippocampal regions in PD with and without dementia have been reported in a few studies that found atrophy in the hippocampal head,^13,14^ CA1-4, DG, subiculum, the whole hippocampus, and ERC in association with poorer memory functions.^2,15–18^

The etiology of CI in PD is not known in detail, but the neuropathology is ultimately heterogeneous involving both cortical Lewy bodies containing α-synuclein and amyloid β (Aβ) plaques.^19^ Neuropathological changes in the hippocampus have been shown in postmortem PD brains with and without dementia, including Lewy neurites, especially in CA2 (refs. 20,21), Lewy neurites, Lewy bodies, and neurofibrillary tangles in PRC and ERC^22^ in addition to Aβ deposition in ERC.^21^

Aβ, tau, and α-synuclein neuropathologies are reflected as neurochemical changes in the cerebrospinal fluid (CSF), and CSF Aβ42, total tau, and phosphorylated tau have been established as biomarkers for AD diagnosis.^23^ On a group level, amyloid species (Aβ42, 40, and 38) are lower than in controls and associated with CI in PD.^24–29^ In most studies, tau levels are reduced or unchanged in PD without dementia.^25,29^ CSF total α-synuclein (t-α-syn) is reduced or unchanged in PD,^25,29^ but its association with CI is not yet clarified.^27–31^

In AD and normal controls (NCs), links between brain atrophy and CSF biomarkers have been reported, supporting the hypothesis that the biomarkers reflect regional or global neurotoxicity related to protein dysmetabolism.^8^ Studies in AD have found associations between lower CSF Aβ42 and CA1 deformation, and hippocampal atrophy and higher T- and phosphorylated tau, and hippocampal, parahippocampal, and ERC atrophy.^8,32^ However, very few studies have assessed the relationship between CSF biomarkers and atrophy in PD. Aβ species and total tau have been associated with ventricular enlargement, but not with hippocampus volume.^33^ Associations between lower Aβ42 and higher tau and frontal, parietal and temporal cortical atrophy were found in PD with dementia,^34^ and lower Aβ42 in non-demented PD patients was prospectively associated with parietal and occipital thinning.^26^ An association between low t-α-syn and frontal cortical thinning in PD without dementia has also been reported.^31^ To our knowledge, no other study has evaluated the hippocampal subfields in relation to CSF biomarkers in PD.

The aims of this study were to measure structural changes of hippocampal subfields and subhippocampal regions and explore possible associations with CSF biomarkers and neuropsychological functions in early, non-demented PD patients with and without MCI. We hypothesized that PD patients have smaller hippocampal subfield volumes compared with NC, and that these changes are associated to CSF biomarkers of neurodegeneration as well as to learning, memory, and visuospatial functions.

## Results

### Comparisons of descriptive and MRI subfields data between PD and NC groups

Descriptive data and CSF measurements of the study participants are presented in Table 1. The PD patients were in early stages with mild to moderate motor symptoms, 13 cases had normal cognition, 5 had subjective CI (SCI), and 15 had MCI, and all had Aβ42 levels above in-house diagnostic cutoff for AD. The PD and NC groups were similar regarding age, education, and distribution of gender. Mini-Mental State Exam was significantly lower in PD compared with NC.

Table 2 presents the MRI volume and thickness data in PD and NC. Total hippocampus, presubiculum, subiculum, CA2–3, CA4-DG, and hippocampal tail were significantly smaller in the PD group, but after adjusting for multiple comparisons only the subiculum and hippocampal tail remained significantly smaller. Before correction for multiple comparisons, none of the 34 cortical thickness variables were significantly thinner in the PD patients compared with the NC group (data not shown).

### Relationship between the MRI subfields data, CSF biomarkers, and cognition in the PD group

Associations between CSF biomarkers and MRI variables in the PD group are shown in Table 3. Lower CSF Aβ38 and 42 were predictors for thinner PRC; a decrease in 2 s.d. of Aβ38 (1550.2 ng/l) and Aβ42 (309.2 ng/l) corresponded to a decrease in PRC thickness of 0.25 and 0.23 mm, respectively. There were no other significant relationships, and no association between Aβ42 and PRC in the NC group (*P*=0.558).

Finally, we assessed the MRI variables and CSF biomarkers as predictors of the neuropsychological tests (Table 4). Lower Aβ42, and smaller presubiculum and subiculum in combination with lower Aβ42 predicted poorer verbal learning and delayed verbal recall; 2 s.d. decrease in Aβ42 (312.98 ng/l), presubiculum (0.184‰ of intracranial volume (ICV)), and subiculum (0.211‰ of ICV) corresponded to a decrease in verbal learning of 10.02, 8.41, and 8.13 points, and a decrease in delayed verbal recall of 1.88, 1.94, and 2.07 points, respectively. The regression models with Aβ42 and presubiculum (subiculum) explained 71.0% (69.5%) and 63.4% (63.6%) of the spread of verbal learning and delayed verbal recall, respectively. The combination of CSF and MRI biomarkers were better predictors of cognition than considered separately. Smaller total hippocampus, presubiculum, and subiculum predicted poorer visuospatial copying; 2 s.d. decrease in total hippocampus (1.397‰ of ICV), presubiculum, and subiculum corresponded to a decrease in visuospatial copying of 7.91, 6.21, and 7.36 points, respectively. Thinner PRC and lower Aβ 38 and 40 predicted poorer delayed visual reproduction separately; 2 s.d. decrease in PRC (0.507 mm), Aβ38 (1557.17 ng/l), and 40 (3567.48 ng/l) corresponded to a decrease in delayed visual reproduction of 5.78, 4.67, and 3.57 points, respectively, but this was no longer significant when combining PRC with Aβ38 or Aβ40 in the same regression analysis. Lower Aβ42 only predicted poorer response inhibition, and no MRI variables predicted executive functions.

## Discussion

The present study is the first study to assess hippocampal subfields and subhippocampal regions in relation to CSF biomarkers in PD patients, proposing Aβ species as early predictors of PRC thickness and as predictors of CI in conjunction with hippocampal subfields. It confirms selective vulnerability of the hippocampal area in early PD patients without dementia. Specifically, we found that subiculum and the hippocampal tail were significantly smaller in PD compared with NC. When exploring the association with cognitive domains in the PD group, we found that smaller subiculum and presubiculum predicted poorer verbal learning and delayed verbal recall, and together with Aβ42, they predicted cognition even better. Lower total hippocampus, presubiculum, and subiculum volumes were associated with poorer visuospatial copying. None of the hippocampal subfields showed associations with the CSF biomarkers. Our findings are in line with studies in primate models and patients with amnestic MCI that have shown associations between hippocampus, subiculum, and presubiculum, and memory and visuospatial functions.^5,6,9,11^

One earlier study in PD using the same subfield segmentation technique found volume loss in CA2–3, CA4-DG, subiculum, and the whole hippocampus, and this correlated with verbal learning.^15^ This is partly in line with our study, and the discrepancies may be due to different PD sample types. For example, our sample had shorter disease duration, fewer with MCI, no one had hallucinations, and there were partly different neuropsychological tests.

Another study in early PD with and without CI found associations between radial distances as a measure of atrophy in CA1, CA3, and subiculum, and poorer verbal recall and recognition, suggesting that these functions are impaired at least partly due to structural hippocampal changes.^16^ They did not find any significant associations between CSF amyloid or tau species and hippocampal radial distance thickness.^33^ The discrepancies with our study may be due to the use of different segmentation techniques, radial distance mapping, and neuropsychological tests.

Even though PRC thickness was not different in PD compared with NC, lower Aβ38 and 42 were significant predictors for thinner PRC in the PD group only. Both lower Aβ38, 40, and thinner PRC predicted poorer delayed visual reproduction, but these associations disappeared when analyzed together. This may indicate that there is a relationship between Aβ species and PRC and delayed visual reproduction, but the findings are not consistent and more research are needed with larger sample sizes to confirm the result. To our knowledge, this is the first study to assess PRC thickness in PD.

PRC is one of the first areas affected by neurofibrillary tangles in pre-dementia AD,^35^ and is also affected in PD.^22^ Aβ may contribute to hyperphosphorylation of tau, which is the main component of neurofibrillary tangles.^36^ PRC have been linked to visual recognition memory,^5^ and CSF Aβ42, visual recognition memory, and PRC are all affected early in patients with amnestic MCI and AD.^10,12,37^ A study in early PD found correlation between low CSF Aβ42 and poorer delayed visual pattern recognition memory in early PD.^28^ On the basis of this and our findings that lower Aβ42 and 38 are predictors for thinner PRC in early PD, we hypothesize that Aβ pathology leads to thinning of PRC, possibly through development of neurofibrillary tangles, and subsequently impaired visual recognition memory. As the present study has a low number of cases with potential bias possibilities, it is important to explore this theory in a larger study population and also include tests of visual recognition memory.

A recent amyloid PET study in cognitively normal elderly showed that cortical Aβ load was related to functional MRI disruption of functional connectivity of the PRC, supporting the hypothesis that dysfunction in PRC may be a very early sign of cortical amyloid deposition and predict memory impairment and dementia.^38^ However, longitudinal follow-up is needed to test this hypothesis in more detail.

As hippocampal subfields and subhippocampal regions are affected and related to poorer memory functions and CSF Aβ42 and T- and phosphorylated tau in MCI and AD,^8–12,32^ our findings are not unique to early PD, but may reflect concomitant AD or at least shared Aβ and tau pathology.

ERC has been reported to be smaller in PD and is associated with poorer learning and memory.^2,17,18^ We found no such association and the discrepancy may be due to longer disease duration in other studies that most of the patients included already had MCI or dementia or also different neuropsychological tests used in these studies.

Although α-synuclein is a synapse protein and a major component of Lewy bodies, the role in CI is unclear,^27–31^ but a possible enhancing interaction with Aβ has been proposed.^39,40^ We found no association between t-α-syn CSF levels and cognition or hippocampal subfield atrophy. More studies are needed to explore the role of t-α-syn and cognition, and CSF studies should measure other α-synuclein species as well.

There was no cortical gray matter thinning in other brain regions, suggesting that differential hippocampal atrophy precedes gray matter changes in early PD. This is in line with another study describing hippocampal atrophy as a biomarker of initial CI in PD.^4^

This is the first study to assess hippocampal subfields and subhippocampal regions with 3 T MRI in relation to CSF biomarkers and cognition in early PD patients. Another strength of this study is CSF t-α-syn measurement in addition to the traditional biomarkers.

3 -T MRI high-field strength employed here allows more detailed imaging of hippocampal substructure. All segmentations were manually inspected, and the image and segmentation quality and consistency were considered good. The segmentation technique was automated, and whether manual segmentation is superior to automated is debated,^41,42^ but we only included the larger hippocampal structures with the best Dice overlap coefficients with manual segmentation.^41^ Anatomical subfields differences may arise due to different segmentation techniques, and a unified segmentation protocol is needed.

Another limitation of the study is the low number of participants. Power calculations for the hippocampal subfields to detect a difference of 7.5% between PD and NC, based on a power of 80%, a significant alpha of 0.05 and s.d.’s for the different hippocampal subfields available from another study,^15^ required 19–41 subjects in each group. Power calculations for subiculum and presubiculum only demanded more participants than in our study. This suggests that the number of participants is adequate to see the most relevant differences. The cross-sectional design is another limitation, and subsequent longitudinal data from this study might inform about the relation between progression of CSF biomarkers, atrophy, and cognition.

In conclusion, regional hippocampal atrophy is present early in PD patients and related to impairments in verbal learning and memory and visuospatial functioning, and probably connected to Aβ pathology. The perirhinal cortex might be the first affected area in PD contributing to impairment of visuospatial memory.

## Materials and methods

### Subjects

The patients in this cross-sectional study were consecutively recruited from a university hospital based neurological outpatient clinic from 2011 to 2014 (ref. 29).

Thirty-three patients diagnosed with PD according to the UK Parkinson’s Disease Society Brain Bank clinical diagnostic criteria,^43^ Hoehn and Yahr stage <3 and disease duration ⩽6 years were included. Neurological examination and diagnosis, the unified PD rating scale part III motor examination and Hoehn and Yahr staging were performed by trained research physicians and movement disorder specialists. All PD patients had pathological ioflupane (^123^I) single-photon emission computed tomography (DaTSCAN) to support the clinical diagnosis of PD. The 15-item Geriatric Depression Scale was used to assess depression. Total daily levodopa equivalent dose was calculated.^44^ Exclusion criteria were dementia, and other severe somatic or psychiatric comorbidity that could contribute to CI, including cancer under treatment, ischemic stroke, drug abuse, solvent exposure, and moderate to severe depression.

Cognitive screening tests were performed in all PD patients and used to categorize the patients into groups of no CI, SCI, MCI, or dementia (exclusion criterion) as described earlier.^45^ Associations between hippocampal subfields and neuropsychological tests were explored in 30 patients using raw scores as previously described;^29^ verbal learning and delayed recall (number of learned words of a list of 15 words over five trials and number of words recalled after 30 min) were measured by Rey Auditory Verbal Learning Test. Visuospatial ability and delayed visual reproduction after 30 min were measured by Rey Complex Figure Test. Aspects of executive functions were assessed by tests measuring divided attention (Trail-Making Test-B), response inhibition (Stroop Color Word), and verbal fluency (Controlled Oral Word Association Test, COWAT). High scores on these tests represent good achievement, except for Trail Making Test-B and Stroop Color Word where high scores represent poor performance.

Thirty-two neurologically healthy NCs were recruited from volunteers, spouses, or relatives of participating patients (*n*=16) and from subjects undergoing elective orthopedic surgery in spinal anesthesia (*n*=16). Inclusion criteria were as previously described^29^ normal cognition based on Mini-Mental State Exam ⩾28, Rey Auditory Verbal Learning Test delayed recall, Trail Making Test-B, and Controlled Oral Word Association Test. Exclusion criteria were as for PD, in addition to relevant subjective cognitive memory symptoms, PD and AD.

Written informed consent was obtained from all participants. The study was approved by the South-Eastern Norway ethical committee for medical research 24 February 2011, last approved change 12 May 2014, approval number 2011/99.

### Cerebrospinal fluid analysis

Thirty-one PD and 23 NC had lumbar puncture. Concentrations of Aβ42, total tau, and phosphorylated tau were analyzed with commercially available enzyme-linked immunosorbent assay kits (Fujirebio Europe, previously Innogenetics, Gent, Belgium). In PD patients, Aβ38, 40, and t-α-syn levels were determined by electrochemiluminescence using anti-Aß 6E10 detection antibody (Meso Scale Diagnostics, Rockville, MD, USA). As red blood cells contain α-synuclein antigenicity, a cutoff value of 350 ng/ml for hemoglobin was determined, and samples with higher hemoglobin concentrations were excluded from the further analysis as previously recommended,^46^ reducing the number of cases to 24 PD for this sub-analysis.

### MRI acquisition, segmentations, and analyses

All participants had Philips Achieva 3-T MRI scanning (Philips Medical Systems, Best, The Netherlands). A single three-dimensional turbo field echo sequence was acquired for morphometric analysis with the following sequence parameters: repetition time/echo time/inversion time/flip angle=4.5 ms/2.2 ms/853 ms/8°, matric=256×213, 170 slices, thickness 1.2 mm, in-plane resolution of 1×1.2 mm. Cortical reconstruction and volumetric segmentation were performed with the FreeSurfer image analysis suite version 5.3.0 (http://surfer.nmr.mgh.harvard.edu/) as previously described.^12^ This includes segmentation of the subcortical white matter and deep gray matter volumetric structures,^47^ and parcellation of the cortical surface according to a previously published parcellation scheme.^48^ This labels cortical sulci and gyri, and thickness values are calculated in the regions of interest (ROI). The thickness values of the ERC and PRC were calculated using methods based on ultrahigh resolution *ex vivo* applied to *in vivo* MRI, as implemented in FreeSurfer.^49,50^ Further, an automated procedure for segmentation of the subfields of the hippocampus, as implemented in FreeSurfer, was employed.^41^ From the hippocampal segmentation, we chose to analyze volume of the following subregions; subiculum, presubiculum, cornu ammonis sector 1 (CA1) separately, sector 2 and 3 combined (CA2–3), sector 4 combined with the dentate gyrus (CA4-DG), and the hippocampal tail, the posterior part of hippocampus that cannot be segmented into the different subfields.^41^ In addition, we analyzed the 34 cortical thickness regions of interests^48^ to differentiate this to hippocampal changes.

### Statistical analysis

Statistical analyses were performed by using IBM SPSS version 20 (Armonk, NY, USA). Continuous variables of demographical and clinical data were compared by Mann–Whitney *U*-tests, and for categorical variables (gender) *χ* ^2^-test was used. The sum of the left and right side MRI subfield volume variables were calculated and were normalized to ICV by dividing the volume variables by ICV. The average of the left and right side PRC, ERC, and 34 cortical thickness variables, and the normalized subfield volume variables were used as dependent variables in multiple linear regression analyses with group (PD or NC), gender, and age as independent variables. The multiple comparisons were corrected for by Bonferroni. They were further assessed as dependent variables in multiple regression analyses with CSF biomarkers as predictors adjusting for age and gender (independent variables) in the PD group. Finally, we performed multiple regression analyses with neuropsychological tests as dependent variables and MRI variables as predictors adjusting for age, gender, and education. CSF biomarkers, unified PD rating scale part III, and levodopa equivalent dose that proved to be significant predictors of a neuropsychological test were included in the multiple regression analysis together with the MRI variables. *P*-values⩽0.05 were considered significant.

## Figures and Tables

**Table 1:** Descriptive data of the study participants

*Descriptive*	*NC*	*PD*	P*-value*
*N*	32	33	—
Age at MRI (years)	62.9 (10.0) (40–82)	64.9 (7.0) (48–74)	0.533^a^
Gender (male/female (%))	44/56	64/36	0.108^b^
Education (years)	13.5 (2.8) (8–18)	13.1 (3.3) (8–19)	0.663^a^
MMSE score	29.3 (0.7) (28–30)	28.6 (1.6) (22–30)	0.019*^a^
Duration of motor symptoms (years)	—	2.7 (1.5) (1–6)	—
Hoehn and Yahr stage (median)	—	1.5 (1–2.5)	—
UPDRS part III motor score	—	14.4 (6.5) (3–31)	—
Levodopa equivalent doses	—	392.2 (249.4) (0–924)	—
Geriatric depression scale	—	1.2 (1.4) (0–6)	—
*N*	23	31	—
Aβ38 (ng/l)	—	1878.6 (775.1) (819.1–4550.9)	—
Aβ40 (ng/l)	—	5125.4 (1772.9) (2212.2–10512.3)	—
Aβ42 (ng/l)	1007.6 (187.2) (692.0–1290.0)	943.0 (154.6) (707.0–1280.0)	—
T-tau (ng/l)	374.9 (188.1) (154.0–893.0)	271.1 (132.8) (119.0–734.0)	—
P-tau (ng/l)	55.7 (18.3) (37.0–110.0)	45.6 (25.3) (25.0–155.0)	—
*N*	—	24	—
T-α.syn (ng/l)	—	282.6 (74.4) (174.3–476.2)	—

Abbreviations: Aβ, amyloid-β; MRI, magnetic resonance imaging; MMSE, Mini-Mental State Examination; NC, normal controls; PD, Parkinson’s disease patients; P-tau, phosphorylated tau; T-tau, total tau; t-α-syn, total α-synuclein; UPDRS, unified Parkinson’s disease rating scale.

Values are presented as mean (s.d.) (range) unless otherwise stated.

a Mann–Whitney test.

b Pearson *χ* ^2^-test.

*Significant *P*⩽0.05.

**Table 2:** Comparison of MRI variables between NC and PD

*MRI variables*	*NC* *mean (s.d.) (range)*	*PD* *mean (s.d.) (range)*	*NC versus PD* a *Adjusted mean difference (95% CI) (*P*-value)*
Total hippocampus (‰ of ICV)	6.0 (1.0) (4.4–8.2)	5.24 (0.74) (3.7–6.8)	−0.492 (−0.834 to −0.149) (0.006)*
Presubiculum, (‰ of ICV)	0.68 (0.13) (0.46–0.97)	0.58 (0.096) (0.41–0.78)	−0.060 (−0.106 to −0.013) (0.014)*
Subiculum, (‰ of ICV)	0.93 (0.15) (0.69–1.25)	0.81 (0.11) (0.60–1.10)	−0.079 (−0.131 to −0.026) (0.004)**
CA1, (‰ of ICV)	0.49 (0.075) (0.38–0.70)	0.45 (0.056) (0.33–0.60)	−0.021 (−0.050 to −0.008) (0.147)
CA2–3, (‰ of ICV)	1.49 (0.24) (1.15–2.01)	1.33 (0.21) (0.95–1.93)	−0.100 (−0.198 to −0.002) (0.046)*
CA4-DG (‰ of ICV)	0.83 (0.14) (0.62–1.14)	0.74 (0.11) (0.53–1.05)	−0.056 (−0.109 to −0.003) (0.038)*
Hippocampal tail (‰ of ICV)	0.55 (0.10) (0.38–0.76)	0.46 (0.07) (0.32–0.62)	−0.058 (−0.095 to −0.021) (0.002)**
PRC (mm)	3.39 (0.24) (2.9–4.0)	3.45 (0.25) (3.0–4.1)	0.078 (−0.048 to 0.205) (0.220)
ERC (mm)	3.53 (0.25) (3.1–4.2)	3.65 (0.27) (3.0–4.2)	0.116 (−0.019 to 0.251) (0.091)

Abbreviations: CA, cornu ammonis; DG, dentate gyrus; ERC, entorhinal cortex; ICV, intracranial volume; MRI, magnetic resonance imaging; NC, normal controls; PD, Parkinson’s disease patients; PRC, perirhinal cortex.

a Multiple linear regression analysis with MRI as dependent variable, and group (PD or NC), gender, and age as independent variables.

*Significant *P*⩽0.05.

^**^Significant *P*⩽0.0056 after adjusting for multiple comparisons by Bonferroni.

**Table 3:** CSF biomarkers as predictors for MRI variables in the PD group

*MRI variables*	*Aβ38*	*Aβ40*	*Aβ42*	*T-tau*	*P-tau*	*t-α-syn*
Total hippocampus (‰ of ICV)	(0.201)	(0.193)	(0.374)	(0.638)	(0.857)	(0.250)
Presubiculum (‰ of ICV)	(0.511)	(0.413)	(0.726)	(0.930)	(0.574)	(0.587)
Subiculum (‰ of ICV)	(0.909)	(0.797)	(0.579)	(0.470)	(0.339)	(0.973)
CA1, (‰ of ICV)	(0.645)	(0.554)	(0.709)	(0.151)	(0.106)	(0.832)
CA2–3, (‰ of ICV)	(0.537)	(0.470)	(0.532)	(0.741)	(0.715)	(0.250)
CA4-DG (‰ of ICV)	(0.724)	(0.623)	(0.792)	(0.678)	(0.506)	(0.218)
Hippocampal tail (‰ of ICV)	(0.787)	(0.499)	(0.948)	(0.630)	(0.272)	(0.807)
PRC (mm)	0.00016 (0.00003-0.00028) (0.018)*	(0.065)	0.00075 (0.00018–0.00133) (0.012)*	(0.250)	(0.779)	(0.828)
ERC (mm)	(0.774)	(0.990)	(0.176)	(0.520)	(0.074)	(0.497)

Abbreviations: Aβ, amyloid-β; CA, cornu ammonis; CI, confidence interval; DG, dentate gyrus; ERC, entorhinal cortex; ICV, intracranial volume; PD, Parkinson’s disease patients; P-tau, phosphorylated tau; t-α-syn, total α-synuclein; PRC, perirhinal cortex; T-tau, total tau.

*N*=31 (24 for t-α-syn).

Multiple linear regression analysis with MRI as dependent variable and CSF biomarkers, as predictors adjusting for age and gender (independent variables).

Values are presented as adjusted effect (95% CI) (*P*-value).

*Significant *P*≤0.05.

**Table 4:** MRI variables and Aβ42 as predictors of neuropsychological tests in the PD group

*Predictors*	*RAVLT verbal learning*	*RAVLT delayed verbal recall*	*RCFT visuospatial copy*	*RCFT delayed visual reproduction*	*TMT-B divided attention*	*SCW response inhibition*	*COWAT verbal fluency*
Aβ42	0.032 (0.012–0.051) (0.003)*^a^	0.006 (0.000–0.012) (0.049)*^a^	(0.431)^b^	(0.263)^a^	(0.159)^a^	–0.039 (–0.077 to –0.002) (0.040)*^b^	(0.628)^b^
Aβ38	(0.323)^a^	(0.198)^a^	(0.388)^b^	0.003 (0.000–0.007) (0.038)*^a^	(0.342)^a^	(0.143)^b^	(0.649)^b^
Aβ40	(0.281)^a^	(0.182)^a^	(0.569)^b^	0.001 (0.000–0.003) (0.049)*^a^	(0.369)^a^	(0.192)^b^	(0.870)^b^
Total hippocampus	(0.130)^c^	(0.079)^c^	5.66 (1.66–9.66) (0.007)*^b^	(0.815)^d^ (0.814)^e^	(0.573)^a^	(0.404)^f^	(0.845)^b^
Presubiculum	45.70 (12.75–78.64) (0.009)*^c^	10.52 (0.282–20.76) (0.044)*^c^	33.77 (5.54–62.00) (0.021)*^b^	(0.355)^d^ (0.386)^e^	(0.103)^a^	(0.052)^f^	(0.948)^b^
Subiculum	38.55 (7.54–69.57) (0.017)*^c^	9.79 (0.420–19.16) (0.041)*^c^	34.90 (10.27–59.52) (0.007)*^b^	(0.184)^d^ (0.216)^e^	(0.231)^a^	(0.139)^f^	(0.324)^b^
CA1	(0.514)^c^	(0.518)^c^	(0.431)^b^	(0.512)^d^ (0.512)^e^	(0.958)^a^	(0.668)^f^	(0.259)^b^
CA2–3	(0.363)^c^	(0.166)^c^	(0.067)^b^	(0.875)^d^ (0.906)^e^	(0.858)^a^	(0.671)^f^	(0.421)^b^
CA4-DG	(0.350)^c^	(0.136)^c^	(0.071)^b^	(0.873)^d^ (0.923)^e^	(0.776)^a^	(0.855)^f^	(0.389)^b^
Hippocampal tail	(0.058)^c^	(0.108)^c^	(0.255)^b^	(0.745)^d^ (0.894)^e^	(0.943)^a^	(0.294)^f^	(0.384)^b^
PRC	(0.709)^c^	(0.516)^c^	(0.993)^b^	11.41 (2.16–20.66) (0.018)*^a^ (0.079)^d^ (0.052)^e^	(0.370)^a^	(0.504)^f^	(0.591)^b^
ERC	(0.256)^c^	(0.216)^c^	(0.646)^b^	(0.225)^d^ (0.183)^e^	(0.350)^a^	(0.159)^f^	(0.707)^b^

Abbreviations: Aβ, amyloid-β; CA, cornu ammonis; COWAT, Controlled Oral Word Association Test; DG, dentate gyrus; MRI, magnetic resonance imaging; PD, Parkinson’s disease patients; PRC, perirhinal cortex; RAVLT, Rey Auditory Verbal Learning Test; RCFT, Rey Complex Figure Test; SCW, Stroop Color Word; TMT-B, Trail-Making Test-B.

*N*=30 (29 for COWAT).

Multiple linear regression analysis with neuropsychological tests as dependent variables and MRI variables, and CSF biomarkers as predictors adjusting for age, gender, and education (in addition to UPDRS for RCFT visuospatial copy, SCW, and COWAT).

Values are presented as adjusted effect in points on neuropsychological tests (95% CI) (*P*-value).

a Adjusting for age, gender, and education.

b Adjusting for UPDRS, age, gender, and education.

c Adjusting for Aβ42, age, gender, and education.

d Adjusting for Aβ38, age, gender, and education.

e Adjusting for Aβ40, age, gender, and education.

f Adjusting for Aβ42, UPDRS, age, gender, and education. *Significant *P*⩽0.05.
